# Supplementary material for: k-mer manifold approximation and projection for visualizing DNA sequences
Source: Genome Res. 2025 May;35(5):1234–46. doi: 10.1101/gr.279458.124 (PMC12047656; doi:10.1101/gr.279458.124)
Supplement: Supplement 8 [file Supplemental_Data_1.zip › Supplemental_Data_1/Nkx6-1_TCGTGT20NGTT_AC_4/kmap_logo.pdf]

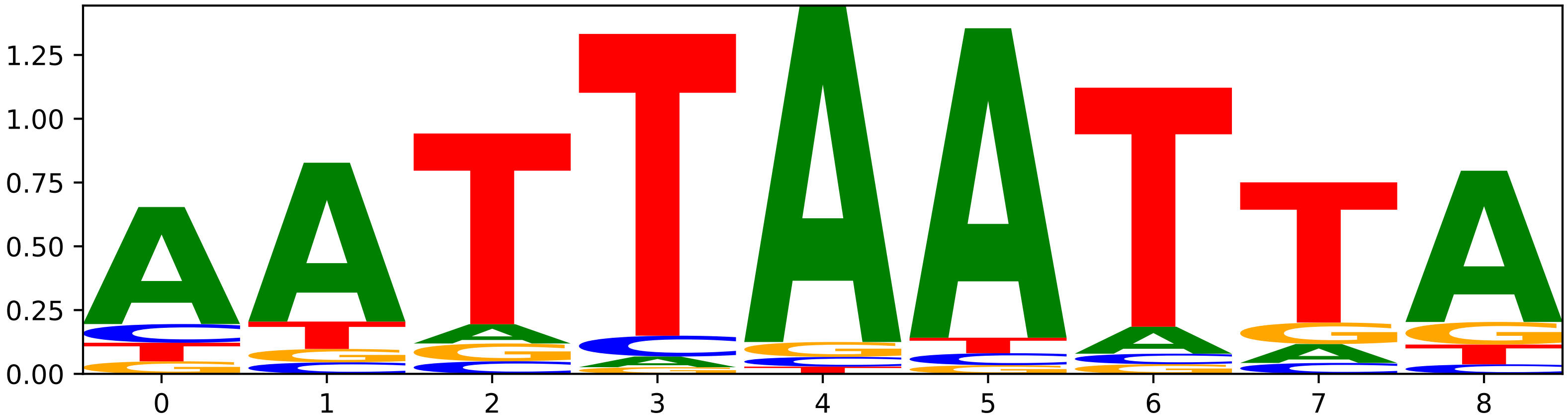

0.00
0.25
0.50
0.75
1.00
1.25
0
1
2
3
4
5
6
7
8
